# An Unusual Cause of Posterior Elbow Impingement: Detachment of a Hypertrophied Posterior Fat Pad

**DOI:** 10.1155/2015/121646

**Published:** 2015-11-03

**Authors:** Daisuke Hamada, Tetsuya Matsuura, Kosuke Sugiura, Tadahiro Higuchi, Naoto Suzue, Tomohiro Goto, Takahiko Tsutsui, Keizo Wada, Shoji Fukuta, Koichi Sairyo

**Affiliations:** Department of Orthopedics, Institute of Biomedical Sciences, Tokushima University Graduate School, Tokushima 770-8503, Japan

## Abstract

We report a case of a 47-year-old woman who developed posterior impingement of the elbow due to detachment of a hypertrophied posterior fat pad. She reported acute left elbow pain after leaning back onto a hard object with her hand and subsequently experienced a “catching” sensation. Comparison with the magnetic resonance images of a normal elbow revealed a hypertrophied posterior fat pad interposed between the olecranon and olecranon fossa in both elbows, with the fat pad in the left elbow located more inferiorly than that in the right elbow. Elbow arthroscopy showed the olecranon fossa covered by the fat pad, a portion of which was detached from the rest of the pad. Debridement of the detached portion was performed until no impingement was evident. Postoperatively, full extension of the elbow did not elicit pain. Clinicians should include this pathology among the differential diagnoses for posterior elbow pain.

## 1. Introduction

Posterior impingement of the elbow is an uncommon disorder in the general population and is typically seen in patients with overuse of the elbow due to specific sports activity, such as overhead throwing, discussed by Moskal et al. [1, 2]. Patients complain of pain at the posterior aspect of the elbow, joint effusion, locking, crepitus, and reduced range of motion (most notably an extension deficit), which occur through a combination of repeated hyperextension, valgus, and supination of the elbow that results in mechanical abutment of bony or soft tissues in the posterior fossa. The common causes of posterior impingement include loose bodies, olecranon stress fracture, and a thickened synovial fold.

We report here an unusual case of posterior impingement of the elbow that was caused by detachment of a hypertrophied posterior fad pad.

## 2. Case Report

**Figure 1:**
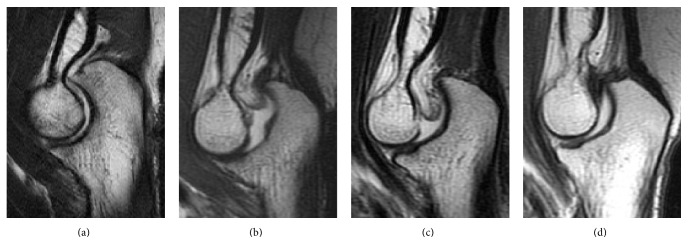
Magnetic resonance images of the elbow. (a) Sagittal T2-weighted image of a normal elbow. Preoperative sagittal T2-weighted images of the (b) right and (c) left side in the present case show a hypertrophied posterior fat pad interposed between the olecranon and olecranon fossa in both elbows. The posterior fat pad in the left elbow is located more inferiorly than that in the right elbow. Postoperative sagittal T2-weighted image of the (d) left elbow shows no posterior fat pad interposed between the olecranon and olecranon fossa.

A 47-year-old woman reported acute left elbow pain after leaning back onto a hard object with her hand while sitting on the ground, a motion wherein axial pressure was applied to the hyperextended elbow. She experienced severe pain along the posterior aspect of the elbow and subsequently a sensation of “catching” during elbow flexion and extension. One year after the injury, she was referred to our hospital. On physical examination, elbow extension of 10° was possible on the asymptomatic right side, whereas it was limited to 5° on the left. Full extension of the elbow elicited tenderness posteriorly, and crepitus was palpable posteriorly with the elbow in full extension. There was no instability of the ulnar collateral ligament and lateral ulnar collateral ligament. Magnetic resonance imaging (MRI) was performed to evaluate the joint space. Comparison with images of a normal elbow revealed a hypertrophied posterior fat pad interposed between the olecranon and olecranon fossa in the left elbow (Figures 1(a) and 1(c)). An MRI of the contralateral side was obtained because we suspected the posterior fat pad had hypertrophied naturally. The posterior fat pad in the right elbow was also hypertrophied (Figure 1(b)). The posterior fat pad in the left elbow was located more inferiorly than that in the right elbow. This constellation of clinical symptoms and imaging findings was compatible with posterior impingement caused by a hypertrophied posterior fat pad.

Use of an anti-inflammatory agent provided no therapeutic benefit. An intraarticular injection of 2% procaine hydrochloride (2 mL) with dexamethasone sodium phosphate (1.65 mg) was administered in the elbow joint. The patient experienced 30% pain relief immediately after injection that lasted for a few days, after which pain with the elbow in extension returned. Surgical intervention was discussed with the patient after nonoperative treatments failed. A left elbow arthroscopy was then performed 2 months following her initial visit.

**Figure 2:**
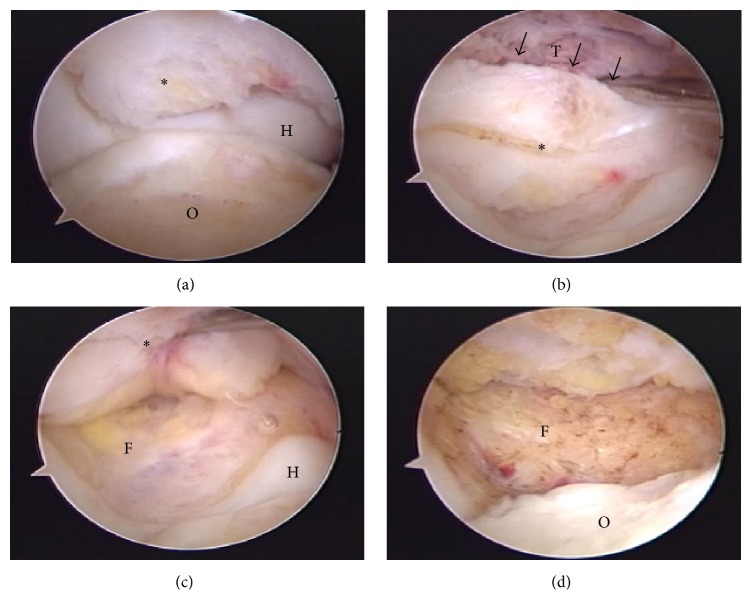
Posterior arthroscopic view of the left elbow. (a) The olecranon fossa is covered by the fat pad (asterisk). (b) The fat pad is partially detached from the rest of the fat pad. (c) The olecranon fossa is visible when the fat pad is pulled upwards. (d) View after debridement of the posterior fossa of the elbow. O: olecranon; H: humerus; T: triceps; F: olecranon fossa. Image views: top: proximal; left: lateral; right: medial; bottom: distal.

During surgery, the patient was placed in the right decubitus position. Standard posterolateral and posteromedial portals were placed. Elbow arthroscopy revealed the olecranon fossa covered by the fat pad, and a portion of the fat pad was noted to be detached from the rest of the hypertrophied fat pad (Figures 2(a) and 2(b)). The olecranon fossa was visible by pulling up the fat pad (Figure 2(c)). After standard arthroscopy of the elbow, debridement of the detached fat pad was performed with a 3.5 mm shaver until no impingement of soft tissue was evident (Figure 2(d)). Postoperative treatment consisted of 1 week of immobilization in a splint, followed by active mobilization under the supervision of a physiotherapist. We did not use any braces. Discontinuing heavy use of the elbow such as carrying heavy loads was recommended for 3 months postoperatively.

At her 1-year clinical follow-up, her elbow was pain-free on full extension and she had no sensation of “catching.” Elbow extension was not restricted compared with the contralateral side. Crepitus was not palpable posteriorly with the elbow in full extension. MRI revealed that the posterior fat pad was not interposed between the olecranon and olecranon fossa in the left elbow (Figure 1(d)).

## 3. Discussion

The differential diagnosis for posterior elbow pain includes loose bodies, olecranon stress fracture, and a thickened synovial fold. In the present case, loose bodies and olecranon stress fracture were absent, and arthroscopy revealed no thickened synovial fold. Thus, this report appears to be the first documented case of posterior elbow impingement caused by detachment of a hypertrophied posterior fat pad.

**Figure 3:**
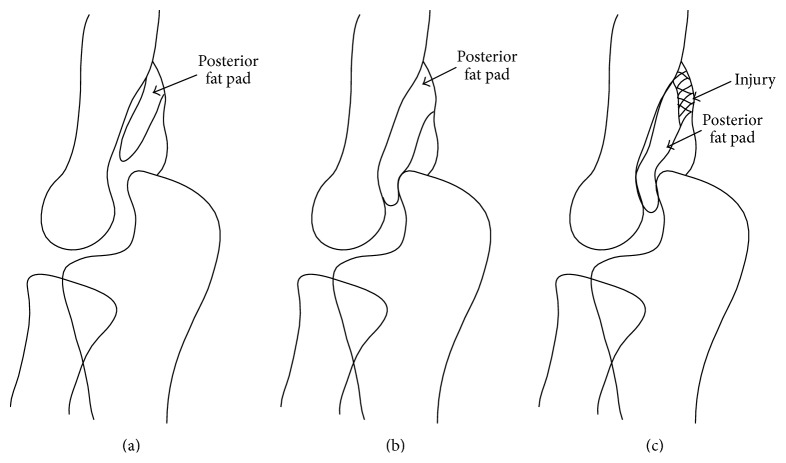
Schematics of the posterior fat pad. (a) Posterior fat pad at the olecranon fossa in the normal elbow. (b) The hypertrophied posterior fat pad in the present case was interposed between the olecranon and olecranon fossa. (c) The posterior fat pad, hanging low at the triceps, was evident in the left elbow in the present case.

The posterior fat pad is extrasynovial and invested by capsular leaflets. The major or deep capsular leaflet is thick and attaches along the articular cartilage margins on a line between the epicondyles and just distal to the olecranon fossa. The superficial leaflet covering the fat pad is only a very thin membranous continuation of the fibrous capsule and it firmly interlaces with the periosteum around the margins of the olecranon fossa [3]. While individual differences in fat pad size are thought to exist, no exact criteria for defining a hypertrophied fat pad have been established. However, as the posterior fat pad is typically thin and normally stays at the olecranon fossa (Figure 3(a)), we attributed the impingement to the location of the hypertrophied fat pad in the present case, namely, interposition between the olecranon and olecranon fossa (Figure 3(b)). In this patient, it was thought that the posterior fat pad had hypertrophied naturally since MRI showed a similar hypertrophied posterior fat pad on the contralateral side.

During extension, the posterior fat pad is highly mobile, whereas during flexion it is pressed into the olecranon fossa by the triceps brachii tendon and the anconeus muscle [4]. We speculated the injury mechanism in the present case to be forcible detachment of the hypertrophied fat pad from the triceps brachii when the patient leant back on her hands on the ground with the elbow in hyperextension. The detachment suggested impingement between the olecranon and olecranon fossa during extension (Figure 3(c)), and this was supported by the arthroscopic findings of detachment of the fat pad from the triceps. Posterior impingement may result from ligamentous instability of the elbow, such as in cases of ulnar collateral ligament or lateral ulnar collateral ligament insufficiency. However, our patient showed no instability of these ligaments.

Treatment for posterior impingement starts with conservative measures such as physiotherapy and nonsteroidal anti-inflammatory drugs combined with rest, ice, compression, and elevation. Steroid injections can sometimes provide some degree of pain relief, especially if the impingement is due to soft tissue swelling; however, conservative therapy may fail to adequately provide long-term symptomatic relief. If conservative treatment fails, arthroscopic intervention with resection of the hypertrophied fat pad is the definitive treatment of choice. Moreover, arthroscopic resection may eliminate symptoms as well as preventing future recurrence.

In summary, we have presented a unique case of posterior elbow impingement due to detachment of a hypertrophied posterior fat pad. The diagnosis was made based on the presence of pain with the elbow in full extension and arthroscopic findings of a detached hypertrophied posterior fat pad. Since such an elbow may manifest clinically as elbow catching, recognizing this lesion as the underlying cause is important for accurate diagnosis. Clinicians should therefore include this pathology among the differential diagnoses for posterior elbow pain.
